# Modification of Caffeic Acid with Pyrrolidine Enhances Antioxidant Ability by Activating AKT/HO-1 Pathway in Heart

**DOI:** 10.1371/journal.pone.0148545

**Published:** 2016-02-04

**Authors:** Hui-Chun Ku, Shih-Yi Lee, Kai-Chien Yang, Yueh-Hsiung Kuo, Ming-Jai Su

**Affiliations:** 1 Institute of Pharmacology, College of Medicine, National Taiwan University, Taipei, Taiwan; 2 Division of Pulmonary and Critical Care Medicine, Mackay Memorial Hospital, Taipei, Taiwan; 3 Mackay Medicine, Nursing and Management College, Taipei, Taiwan; 4 Department of Chinese Pharmaceutical Sciences and Chinese Medicine Resources, China Medical University, Taichung, Taiwan; 5 Department of Biotechnology, Asia University, Taichung, Taiwan; Chang Gung University, TAIWAN

## Abstract

Overproduction of free radicals during ischemia/reperfusion (I/R) injury leads to an interest in using antioxidant therapy. Activating an endogenous antioxidant signaling pathway is more important due to the fact that the free radical scavenging behavior in vitro does not always correlate with a cytoprotection effect in vivo. Caffeic acid (CA), an antioxidant, is a major phenolic constituent in nature. Pyrrolidinyl caffeamide (PLCA), a derivative of CA, was compared with CA for their antioxidant and cytoprotective effects. Our results indicate that CA and PLCA exert the same ability to scavenge DPPH in vitro. In response to myocardial I/R stress, PLCA was shown to attenuate lipid peroxydation and troponin release more than CA. These responses were accompanied with a prominent elevation in AKT and HO-1 expression and a preservation of mnSOD expression and catalase activity. PLCA also improved cell viability and alleviated the intracellular ROS level more than CA in cardiomyocytes exposed to H_2_O_2_. When inhibiting the AKT or HO-1 pathways, PLCA lost its ability to recover mnSOD expression and catalase activity to counteract with oxidative stress, suggesting AKT/HO-1 pathway activation by PLCA plays an important role. In addition, inhibition of AKT signaling further abolished HO-1 activity, while inhibition of HO-1 signaling attenuated AKT expression, indicating cross-talk between the AKT and HO-1 pathways. These protective effects may contribute to the cardiac function improvement by PLCA. These findings provide new insight into therapeutic approaches using a modified natural compound against oxidative stress from myocardial injuries.

## Introduction

Reactive oxygen species (ROS) are continuously generated in the heart as a by-product of cellular respiration and metabolism [1]. Low concentrations of ROS participate in several physiological processes, such as cell signaling and gene expression [1]. Excessive ROS production results in cell damage and is one of the known contributors to the pathogenesis of cardiovascular disease [2, 3]. Cellular redox balance is tightly regulated, while ROS production is eliminated by endogenous antioxidant enzymes [4]. The formation of ROS is accomplished by the conversion of oxygen to a superoxide anion, which has a very short half-life and is rapidly converted to the less-reactive hydrogen peroxide (H_2_O_2_) by superoxide dismutases (SOD), and then H_2_O_2_ is continuously decomposed into H_2_O and oxygen by catalase [1]. A burst of ROS occurring during myocardial ischemia/reperfusion (I/R) stress [4, 5], which results in the exhaustion of endogenous free radical scavenger enzymes [4, 5]. Targeting ROS with various antioxidant agents or upregulation of endogenous free radical scavengers has been shown to alleviate myocardial I/R injury [2].

Heme oxygenate (HO), a rate-limiting enzyme for heme degradation [6], is a regulator of oxidative stress and plays a critical role on the cardiovascular system [7, 8]. HO catabolizes the oxidation of heme into biliverdin, which is rapidly converted to bilirubin by biliverdin reductase [9]. Bilirubin is then converted back into biliverdin through the actions of ROS [10]. This cycle allows for the neutralization of ROS, which is considered as one of the antioxidant effects of HO-1 [11]. There are two isoforms of HO in the heart: HO-1 and HO-2 [9]. HO-2 is constitutively expressed under physiological conditions [9]. HO-1 is stress inducible to guard against oxidative insult suggests a countervailing system to oxidative injury [6, 12]. Upregulation of HO-1 shifts the redox state to a reduced state and decreases superoxide formation [13]. Besides antioxidant ability, HO-1 has been shown to exert other biological properties that play important roles on cellular protection including anti-inflammatory and anti-apoptosis abilities [9]. Elevation of the HO-1 level has been shown to be associated with an alleviation of the severity of coronary artery disease in clinics [14]. Compounds that activate HO-1 signaling may act as a potential agent for I/R injury [7, 15–17].

Caffeic acid (CA), a phenolic compound frequently present in our diet, such as fruits, vegetables, herbs and honey [18], has been demonstrated to exhibit a variety of properties including immunomodulatory, anti-inflammatory, and free radical scavenging activity [19, 20]. The catechol ring in CA is largely accountable for the free radical scavenging property. However, the half-life of CA is short [21], and the low bioavailability limits the effectiveness of using CA as a therapeutic agent. Proper modeling of the compound is needed to overcome bioavailability limits in order to evaluate its therapeutic effects. Our previous study demonstrated that pyrrolidinyl caffeamide (PLCA), a synthetic derivative of CA, activated AKT signaling to prevent heart injury from I/R stress and improve cell survival [22]. The protective effect of PLCA is more efficient than CA. Since AKT signaling is involved in regulating the HO-1 pathway [23, 24], whether PLCA activates HO-1 signaling and whether HO-1 signaling contributes to the protective effect is in need of clarification. Therefore, in this study, we set out to investigate the response of PLCA on I/R-induced ROS stress.

## Materials and Methods

### Preparation of PLCA

PLCA was produced from 100 mg caffeic acid dissolved in 1mL N,N-dimethylformamide and 80 μL triethylamine in a two-necked bottle. The solution was then added into 1.2 mol, 5 mL dichloromethane containing pyrrolidine and 1.2 mol benzotriazol-1-yloxytris(dimethylamino)phosphonium hexafluorophosphate to react for 30 minutes in an ice bath, followed by reacting at the room temperature for 2 hours. After the reaction is finished, dichloromethane was removed. The residue was then added into 50 mL water, and extracted by ethyl acetate. The organic phase was then collected, and washed with HCl, NaHCO_3_ and followed by removing water with anhydrous sodium sulfate. After performing a filtration, condensation, and column chromatography, PLCA was obtained.

### Free radical scavenging activity in vitro

The free radical scavenging ability of CA and PLCA was determined by a DPPH scavenging assay as described previously with modifications [25]. Briefly, different concentrations of compounds were added to a DPPH solution, then after 30 min in dark incubation, the decrease in the absorbance was measured at 517 nm. The half maximal inhibitory concentration (IC_50_) was used as an indicating measure of the scavenging activity.

### Experimental model of myocardial I/R injury in vivo

This research was conducted in accordance with the Guide for the Care and Use of Laboratory Animals, published by the US National Institutes of Health (NIH publication no. 85–23, revised 1996), and was approved by the Institutional Animal Care and Use Committee of National Taiwan University, Taiwan. Sprague—Dawley rats (BioLASCO Taiwan, Co., Ltd, Taipei, Taiwan) were used. Eight-week-old male animals were maintained under a 12-h light/dark cycle at a controlled temperature (21 ± 2°C) with free access to food and tap water. Rats were randomly divided into four groups (6 rats in each group), and anesthetized with pentobarbital (75mg/kg, i.p.). After appeared calm, rats were subjected to 1 h coronary artery occlusion, followed by 2 h reperfusion as described previously [26, 27]. A 1 mg/kg dose of either PLCA or CA was administered by intraperitoneal injection 15 min after coronary artery occlusion started. During I/R injury, rats were placed on an electric blanket to maintain body temperature. One third of the animals died due to the severe infarction and was withdrawn from the groups. After 2 hour of reperfusion, cervical dislocation was performed, and the hearts were collected for the protein analysis.

### Determination of Cardiac Function

Cardiac function was estimated in vivo by a pressure—volume catheter (1.9 F; Scisense Instruments, Ontario, Canada), as described previously [28, 29]. The catheter was inserted into the carotid artery and then advanced into the left ventricle for the measurement of pressure and volume. All pressure—volume loop (PV loop) data were analyzed using a cardiac analysis program of PONEMAH Life Sciences Suite from Data Sciences International (DSI Corporate Headquarters, St. Paul, MN, USA). PV loops were recorded, and several parameters were chosen for analysis: maximal slope of the systolic pressure increment (+dP/ dt), maximal slope of the diastolic pressure decrement (−dP/dt), and stroke work (SW). SW is used to assess ventricular function by calculating the area within the PV loop.

### Protein Extraction

Left ventricles were homogenized via an extraction buffer (Thermo Fisher Scientific Inc., IL, USA) containing cocktail protease and phosphatase inhibitor (Sigma, St. Louis, MO, USA) as described previously [26]. The supernatant of homogenate was collected after centrifugation (800×g, 10 min at 4°C), and the protein concentration was determined using a BCA protein assay kit (Thermo Fisher Scientific Inc., IL, USA).

### Determination of troponin concentration

Myocardial injury was determined using a cardiac specific marker, troponin, measured using an ELISA kit (Life Diagnotics Inc., PA, USA).

### Determination of lipid peroxidation

Malondialdehyde, an end product of cell membrane lipid peroxidation, was used as a reliable marker of myocardial cell damage and oxidative stress, and measured using a kit (Cayman Chemicals, MI, USA). Protein was reacted with thiobarbituric acid at 100°C for 1 h. The fluorescence signal was detected at the excitation wavelength of 360 nm and an emission wavelength of 450 nm using a microplate spectrophotometer.

### Determination of catalase activity

Catalase activity was determined by the decomposition of H_2_O_2_ with a kit (Cayman Chemical, Ann Arbor, MI).

### Determination of HO-1 activity

Tissue homogenate or cell lysate was used to measure HO-1 activity. The reaction mixture, containing the sample, rat liver cytosol (source of biliverdin reductase), and 1mM NADPH, was incubated at 37°C for 1 h and protected from light. The amount of bilirubin production was measured using a kit (Sigma Diagnostics, St. Louis, MO) to determine HO-1 activity.

### Determination of myeloperoxidase (MPO) activity

Cardiac tissue homogenate was used to detect MPO activity by using ELISA kit (Santa Cruz Biotechnology, Inc.).

### Cell Culture

HL-1, a mouse atrial cardiomyocyte, is used in this study and obtained from Dr. William C. Claycomb (Louisiana State University Health Sciences Center, New Orleans, LA). HL-1 cells can be serially passaged, maintain the ability to contract and retain differentiated cardiac morphological, biochemical, and electrophysiological properties [30]. The cells were cultured in Claycomb medium supplemented with 10% FBS (Gibco, Scotland, UK), 2 mM L-glutamine (Gibco, Scotland, UK), 0.1 mM norepinephrine and antibiotics (100 μg/ml penicillin and 100 μg/ml streptomycin) at 37°C under a 5% CO_2_ air atmosphere. H_2_O_2_ was added to induce oxidative stress. CA and PLCA (1, 3, 10 μM) were pretreated 1 h before H_2_O_2_ administration. To explore the mechanisms of PLCA, either LY294002 (PI3K inhibitor; 15 μM) or tin protoporphyrin IX (SnPP, HO-1 inhibitor; 5 μM) were incubated 30 min before the addition of PLCA.

### Detection of cell viability

Cell viability was determined by a MTT assay as describe previously [31]. Cells were treated with MTT (3-(4,5-Dimethylthiazol-2-yl)-2,5-diphen​yltetrazoliumbromide) at 0.5 mg/ml. The purple formazan crystals were dissolved in DMSO. Solutions were then loaded into a 96 well plate, and the absorbance was determined on an automated microplate spectrophotometer at 570 nm.

### Detection of ROS production in cardiomyocytes

Intracellular ROS production in cardiomyocytes was labeled with fluorescent dye CM-H2DCFD, as described previously [31], and monitored at 488 nm excitation and 515 nm emission using fluorescence microscopy. Fluorescence intensity was calculated by averaging fluorescence intensity of numerous outlined cells using Imagequant (Molecular Dynamics, Inc., Sunnyvale, CA, USA).

### Western blot

A Western blotting technique was performed on detecting protein expression, following methods as described in a previous study [31]. Protein samples were first denatured for 10 min in boiling sample buffer (31.3 mM Tris-HCl pH 6.8, 25% glycerol, 10% SDS, 10% 2-Mercaptoethanol, 0.00125% bromophenol blue), then separated by SDS-PAGE and transferred to polyvinylidene difluoride membranes (Perkin-Elmer Life Sciences, Boston, MA, USA). The membranes were blocked in 5% fat-free milk dissolved in TBST (Tris/phosphate/saline/Tween) and incubated with the primary antibodies of p-AKT, AKT, mnSOD, HO-1 expression (abCAM, USA) and β-actin (Santa Cruz Biotechnology, CA, USA) overnight at 4°C. The membranes were then washed several times with TBST, followed by further incubation with horseradish peroxidase-conjugated secondary antibodies (Santa Cruz Biotechnology, Inc.) for an hour. After being washed several times, the protein signals were detected with the ECL system (Millipore, Bedford, MA, USA). The blots were scanned and quantified by Imagequant (Molecular Dynamics, Inc., Sunnyvale, CA, USA).

### Statistical Analysis

All values were represented as means ± SE. The results were analyzed using ANOVA, followed by Bonferroni's post hoc tests. P<0.05 was considered as significant difference.

## Results

### Comparison of the antioxidant activity between CA and PLCA in vitro

**Fig 1:**
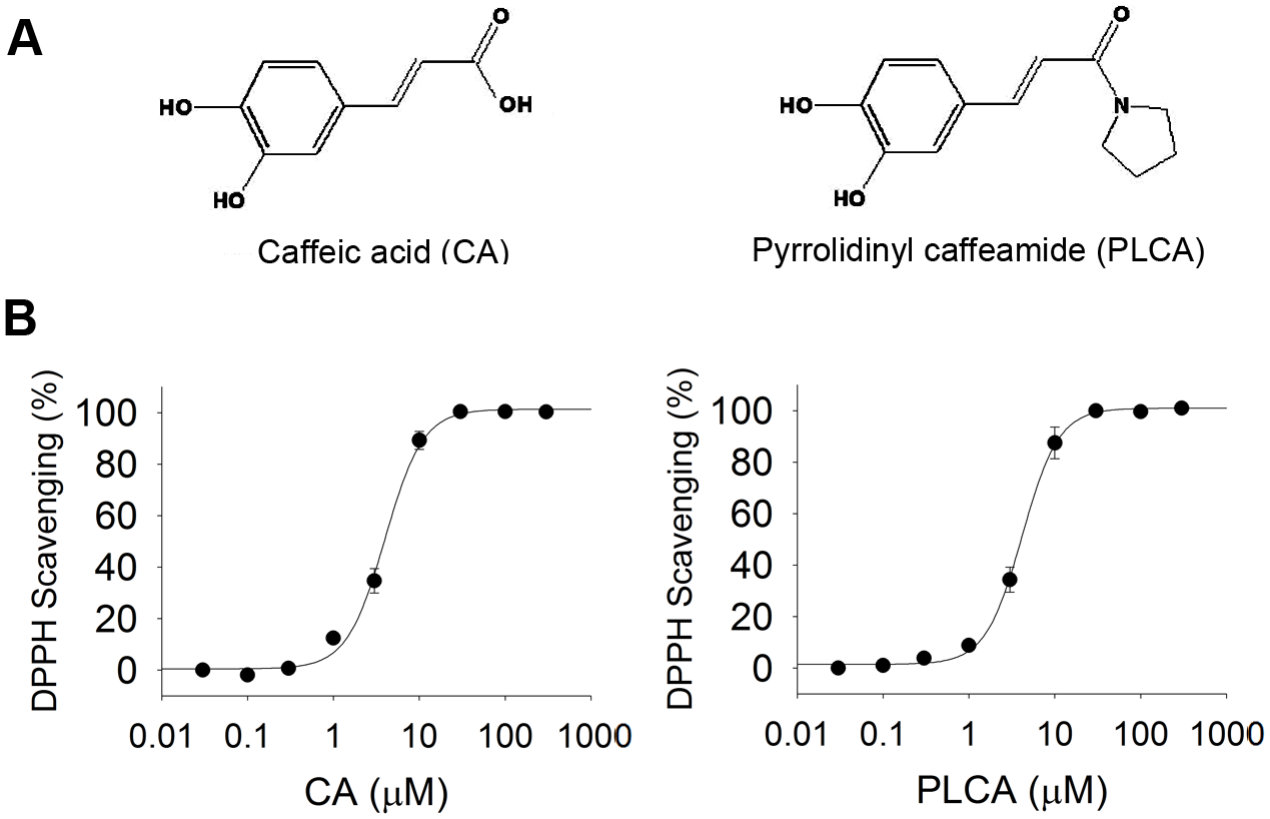
Chemical property of caffeic acid and its derivative. (A) Structure of caffeic acid (CA) and pyrrolidinyl caffeamide (PLCA). (B) DPPH scavenging ability of CA and PLCA. Concentrations of two compound is from 0.03 μM to 300 μM (n = 3).

The structures of CA and PLCA are shown in Fig 1A. The in vitro antioxidant activities (IC_50_) of the two compounds measured using the DPPH assay is shown in Fig 1B. The IC_50_ values of CA and PLCA on DPPH were 4.0 ± 0.2 μM and 4.2 ± 0.1 μM, respectively. There was no significant difference in free radical scavenging ability between the two compounds.

### Comparison of oxidative injury and protein expression between CA and PLCA after rats subjected to myocardial I/R stress

**Fig 2:**
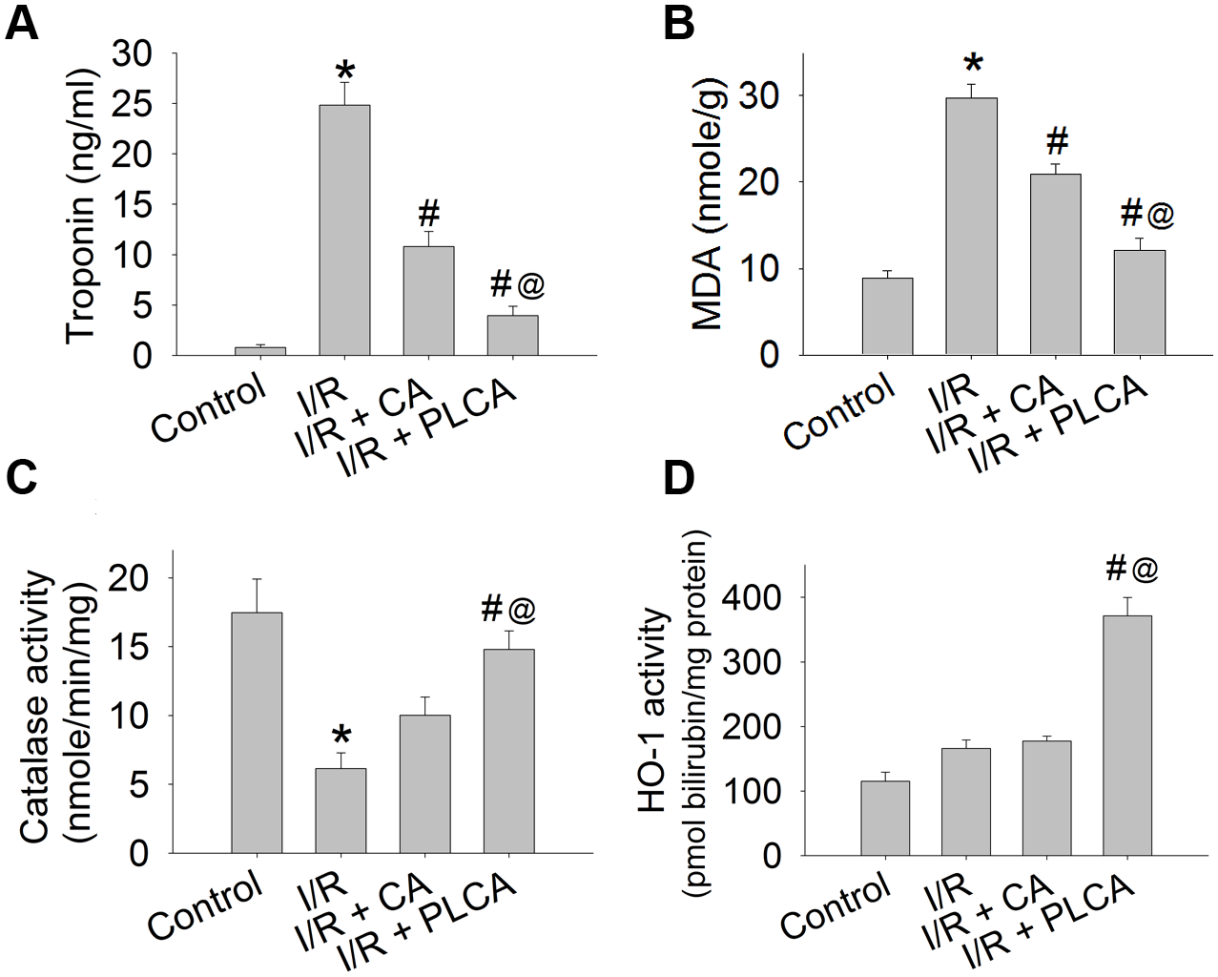
Effect of CA and PLCA on myocardial I/R stress induced oxidative injury and changes of protein activity. Cardiac oxidative injury and protein activity were shown in rats subjected to 1 h coronary artery occlusion and followed by 2 h reperfusion. (A) Troponin concentration, (B) lipid peroxydation, (C) catalase activity, and (D) HO-1 activity were measured. (n = 6) *P < 0.05 vs. control, # < 0.05 vs. IR, @ < 0.05 vs. IR+CA.

**Fig 3:**
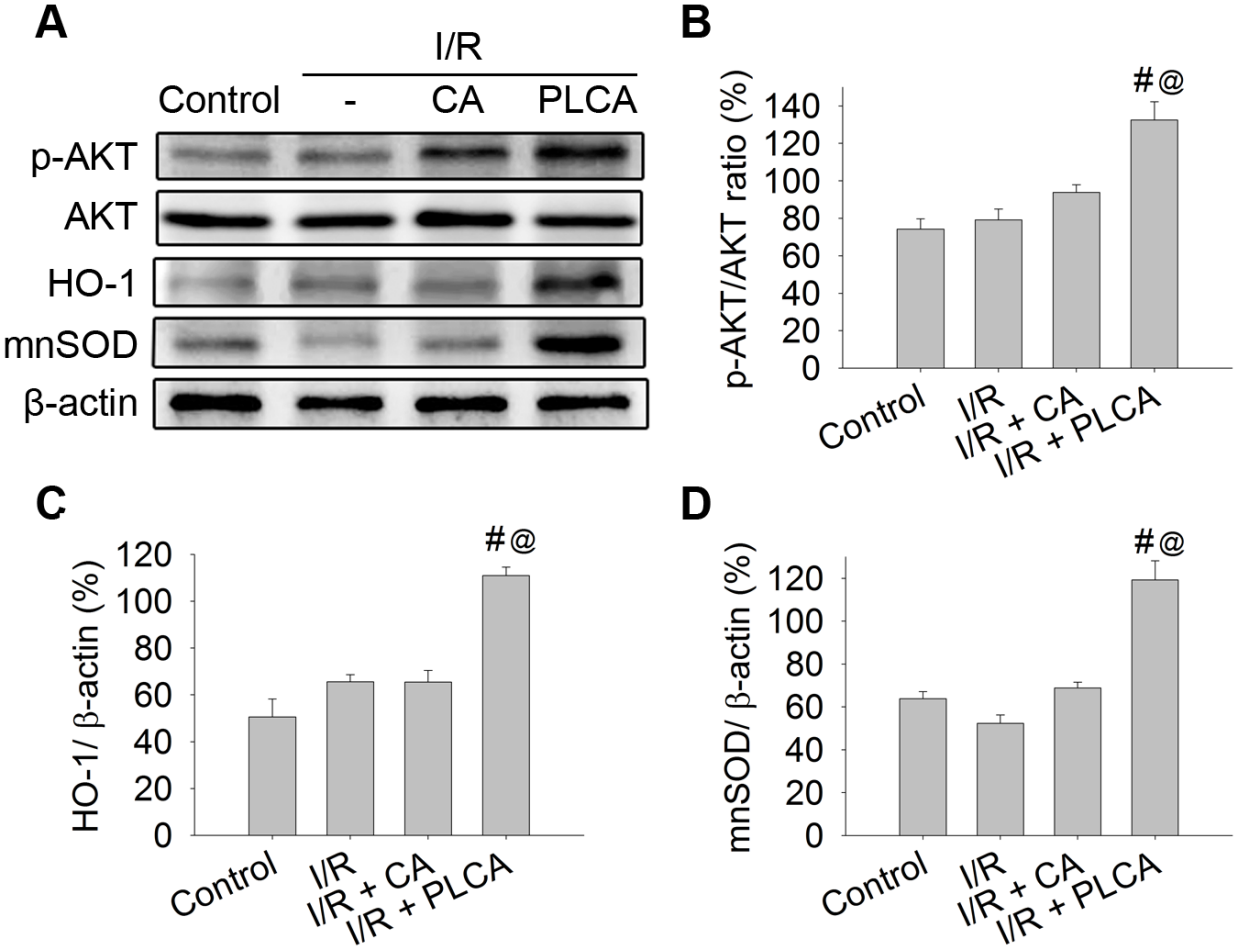
Effect of CA and PLCA on protein expression after rat subjected to myocardial I/R stress. Protein expression was shown in rats subjected to 1 h coronary artery occlusion and followed by 2 h reperfusion. (A) Original western blots were shown. (B) Ratios of p-AKT to AKT, (C) HO-1 to β-actin, (D) mnSOD to β-actin were measured. (n = 4) *P < 0.05 vs. control, # < 0.05 vs. IR, @ < 0.05 vs. IR+CA.

Rats subjected to myocardial I/R stress resulted in the elevation of plasma troponin concentration (Fig 2A), and myocardium lipid peroxidation (Fig 2B) and loss of catalase activity (Fig 2C), which are all indicators of oxidative injury in the heart. The protective effects of CA and PLCA on the heart were compared. PLCA was shown to attenuate troponin and lipid peroxidation and preserve catalase activity more than CA. Moreover, PLCA induced a prominent elevation of HO-1 activity in the heart (Fig 2D), while CA had no significant effect on HO-1 activity. Protein expression was also detected after rats were subjected to myocardial I/R stress (Fig 3A). Only PLCA significantly stimulated p-AKT, HO-1, and mnSOD expression (Fig 3B, 3C and 3D).

### Comparison of the oxidative injury between CA and PLCA in cardiomyocytes exposed to H_2_O_2_

**Fig 4:**
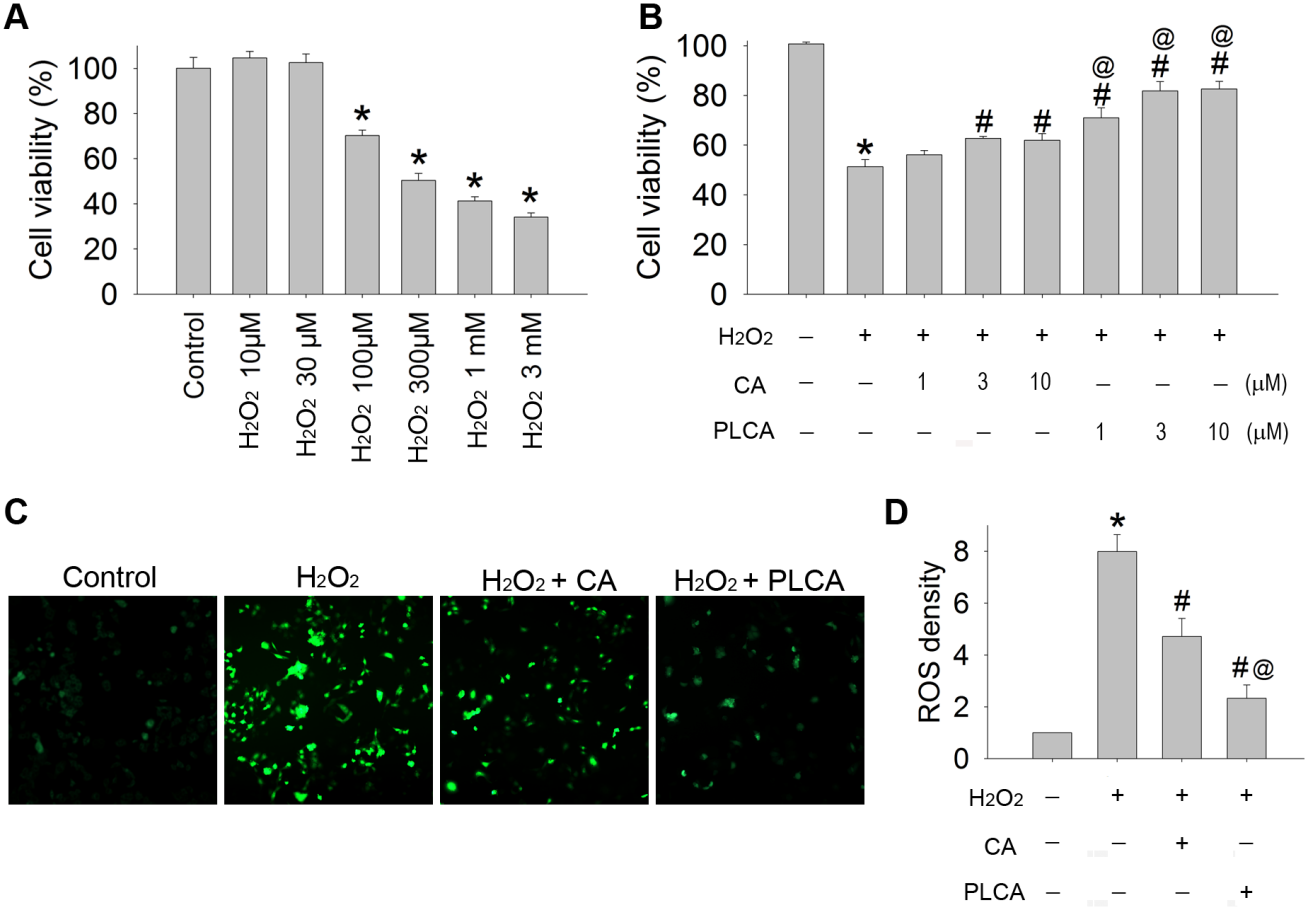
Effect of CA and PLCA on cardiomyocyte exposed to H_2_O_2_. (A) Cell viability was measured in cardiomyocyte exposed to H_2_O_2_ (10 μM ~ 3 mM). (B) Different doses of CA or PLCA was treated 1 h before H_2_O_2_ (300 μM) administration. (C) ROS generation in cardiomyocytes exposed to H_2_O_2_ was detected by CM-H2DCFD, and (D) the results of fluorescence density were also calculated. (n = 4) *P < 0.05 vs. control, # < 0.05 vs. H_2_O_2_, @ <0.05 vs. H_2_O_2_ + CA in same dose.

Cardiomyocytes were exposed to different concentrations of H_2_O_2_, ranging from 10 μM to 3 mM (Fig 4A). H_2_O_2_ decreased cell viability in a concentration-dependent manner. A 300 μM concentration resulted in a 51.3% remnant of cell viability and was chosen for the following experiments. Different concentrations (1, 3, and 10 μM) of CA or PLCA were added 1 h before H_2_O_2_ exposure. Both CA and PLCA treatments were capable of improving cell viability. Administration of both drugs at 3 μM achieved the ceiling effect in protecting cell viability, at 60.9% and 76.1% of the control group, respectively (Fig 4B). The intracellular level of ROS was measured (Fig 4C and 4D). After H_2_O_2_ treatment, the ROS level significantly elevated in cardiomyocytes. Both CA and PLCA treatments before H_2_O_2_ administration resulted in an alleviation of the ROS concentration, but PLCA attenuated the ROS level more than CA in the cardiomyocytes. The ability of a compound to improve cell viability correlated with the alleviation of the intracellular ROS level in cardiomyocytes exposed to H_2_O_2_. Thus, PLCA is more efficient than CA in protecting against H_2_O_2_ induced oxidative injury in cardiomyocytes.

### PLCA protected cardiomyocytes against oxidative injury via the AKT/HO-1 pathway

**Fig 5:**
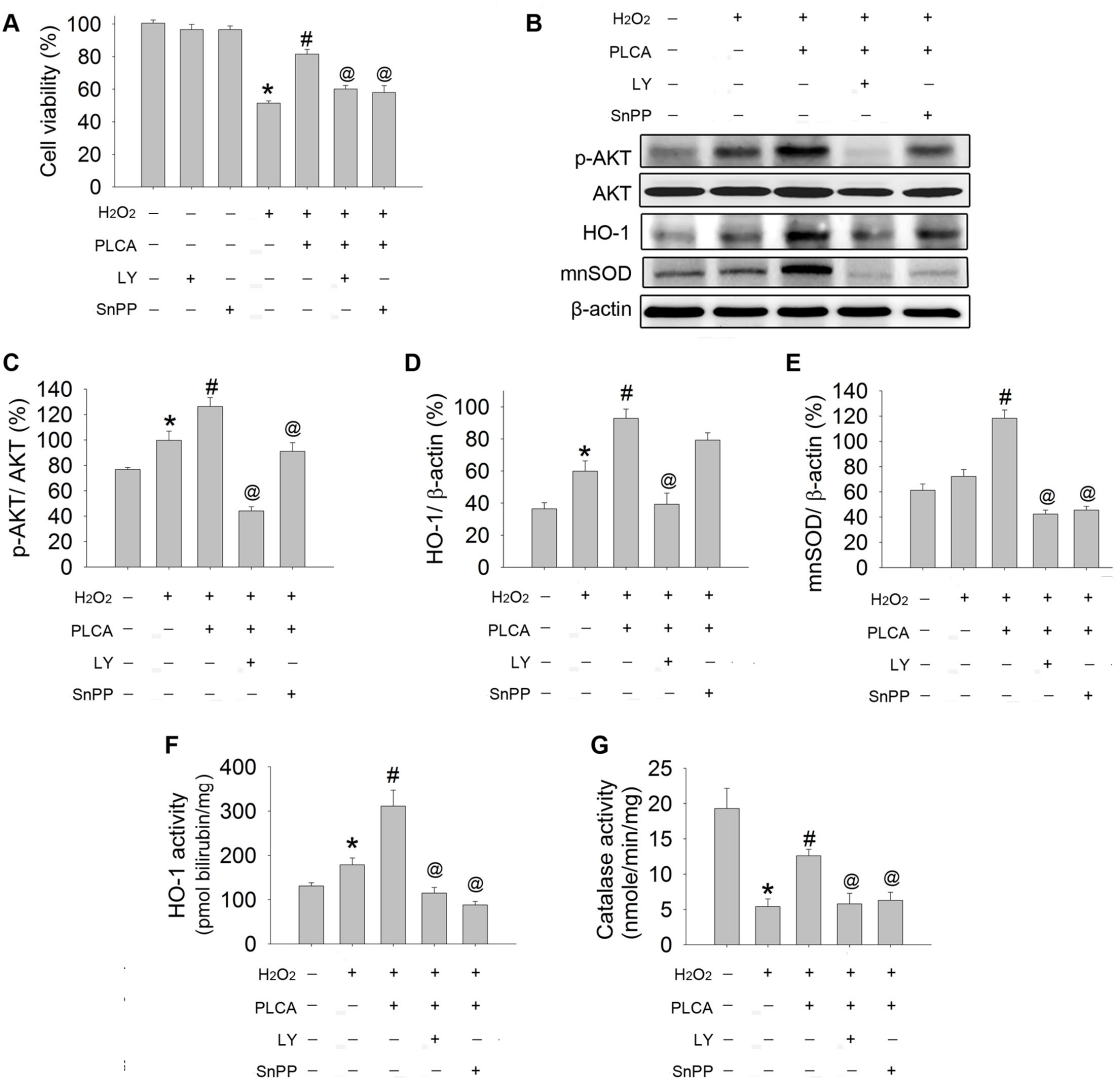
The signaling pathway involved in PLCA against oxidative stress. (A) Cell viability was measured in cardiomyocyte exposed to H_2_O_2_ (300 μM) in the presence or absence of PLCA combination with LY294002 (15 μM; PI3K inhibitor) or SnPP (10 μM; HO-1 inhibitor). (B) Original western blots were reported. (C) Ratios of p-AKT to AKT, (D) ratios of HO-1 to β-actin HO-1 activity, (E) ratios of mnSOD to β-actin, (F) HO-1 activity, and (G) catalase activity were measured. (n = 4) *P < 0.05 vs. control, # < 0.05 vs. H_2_O_2_, @ < 0.05 vs. H_2_O_2_+PLCA.

We then investigated the contributions of AKT and HO-1 to the protective role of PLCA by using LY294002, a PI3K pathway-blocking reagent, and SnPP, a HO-1 inhibitor. Neither LY294002 nor SnPP alone had any effect on cell viability (Fig 5A). However, when PLCA was combined with LY294002 or SnPP, PLCA lost its protective effect on cell viability in cardiomyocytes exposed to H_2_O_2_.

Protein expression or activity was detected in cardiomyocytes after H_2_O_2_ exposure (Fig 5B). H_2_O_2_ resulted in an elevation in AKT phosphorylation, which was further upregulated by PLCA (Fig 5C), along with an enhancement in HO-1 expression and activity, and mnSOD expression (Fig 5D, 5E and 5F), and a preservation in catalase activity (Fig 5G). The combination of LY294002 with PLCA treatment abolished the stimulation effect of PLCA on p-AKT expression in cardiomyocytes exposed to H_2_O_2_, resulting in loss of HO-1 activity, mnSOD expression, and catalase activity. On the other hand, the combination of SnPP with PLCA treatment also abolished the stimulation effect of HO-1 activity in cardiomyocytes exposed to H_2_O_2_, accompanied with loss of mnSOD expression and catalase activity. Interestingly, SnPP also attenuated the increase in AKT phosphorylation by PLCA in cardiomyocytes exposed to H_2_O_2_.

### PLCA improved cardiac function after rats subjected to myocardial I/R stress

**Fig 6:**
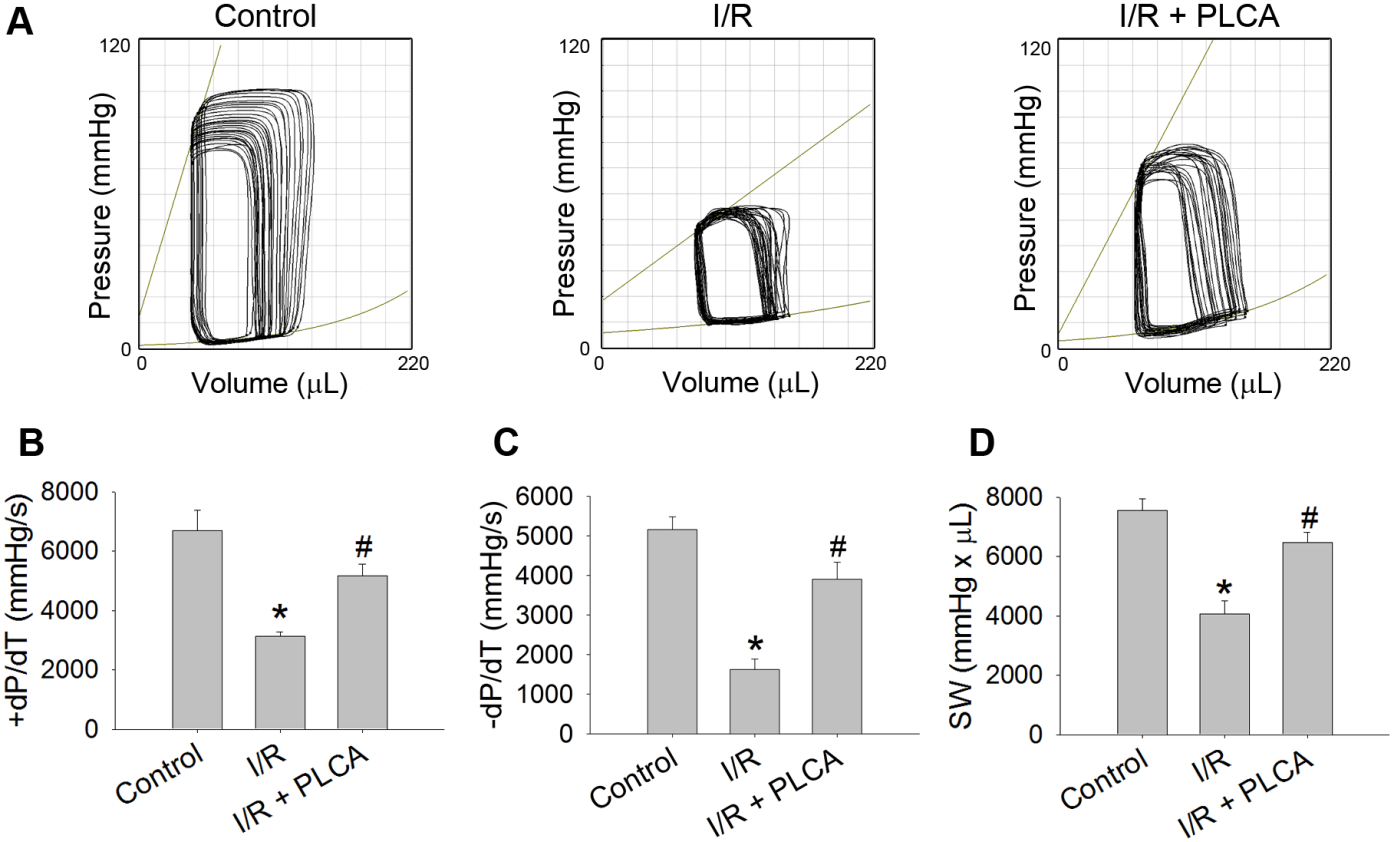
Effect of PLCA on cardiac function after rat subjected to myocardial I/R stress. Cardiac functions were measured by pressure-volume loop (PV loop). (A) Representative PV loop of each groups were shown. (B) Maximal slope of the systolic pressure increment (+dP/ dt), (C) maximal slope of the diastolic pressure decrement (−dP/dt), and (D) stroke work (SW) were measured. (n = 6)*P < 0.05 vs. control, # < 0.05 vs. IR.

The cardiac functions of the rats were measured using PV loops after each rat was subjected to myocardial I/R stress (Fig 6). I/R caused a reduction in the maximal slope of the systolic pressure increment, maximal slope of the diastolic pressure decrement and the stroke work, indicating that cardiac dysfunction had occurred. PLCA treatment recovers cardiac performance after I/R stress in rats.

### PLCA alleviated neutrophil infiltration in myocardial I/R

**Fig 7:**
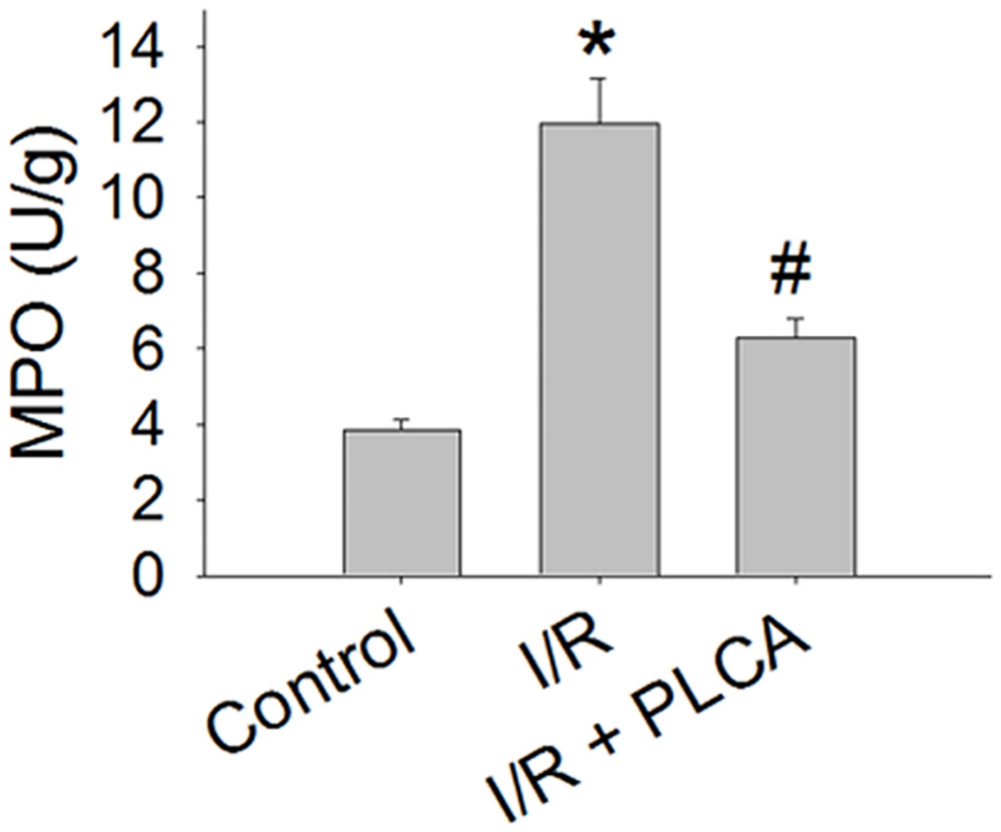
Effect of PLCA on myocardial I/R-induced neutrophil infiltration. Myeloperoxidase (MPO) activity was detected in I/R-injured cardiac tissue. (n = 6) *P < 0.05 vs. control, # < 0.05 vs. IR.

MPO activity, a marker of neutrophil accumulation, was increased in I/R heart, which was attenuated by PLCA (Fig 7).

## Discussion

In this study, we demonstrated that CA and PLCA exert the same ability to scavenge DPPH in vitro. However, PLCA is more efficient than CA in protecting against oxidative stress. In response to myocardial I/R stress, PLCA was shown to attenuate lipid peroxidation and troponin release more than CA, and exhibited a prominent elevation of AKT and HO-1 expression and preservation of mnSOD and catalase activity. PLCA also improved cell viability and alleviated intracellular ROS levels more than CA in cardiomyocytes exposed to H_2_O_2_. When inhibiting the AKT or the HO-1 pathway, PLCA lost its ability to recover mnSOD expression and catalase activity to counteract oxidative stress, suggesting that the AKT/HO-1 pathway is activated by PLCA. These protective effects may contribute to the cardiac function improvement of PLCA.

Overproduction of ROS reacts with fatty acids resulting in formation of lipid peroxidation and inhibition of the mitochondrial electron transport chain which impairs the function and structure of enzyme systems and induces cell death [32]. Deleterious radicals include superoxide, peroxynitrite, hydroxyl radical and H_2_O_2_ [2], which are derived from a variety of sources, including electron transport chain in mitochondria, xanthine oxidase, cytochrome oxidase, and cyclooxygenase [33]. The production of excessive quantities of ROS is the predominate cause of I/R injury [3, 34]. Treatment with antioxidant agents has been successfully tested in several experimental models of I/R; however, clinical studies with antioxidants have thus far yielded disappointing results [2], which may be related to low bioavailability [2]. Modification of natural compound structure with side chain can result in dramatic changes in enzyme activity, such as HO-1 activation [7]. CA modified with amide improves the stability of the compound in rat plasma [35], which leads to prolonged in vivo half-life, allows the compound to remain in circulation, and exerts its effects for a longer period of time, resulting in bioavailability improvement [36]. PLCA with a hydrophobic side chain may enhance bioavailability and induce the endogenous effect. In our study, PLCA was found to improve mnSOD expression and catalase activity, reduce the level of lipid peroxidation and neutrophil infiltration in I/R myocardium. Neutrophil also serves as a potent stimulus for ROS production [37]. Neutrophil activated by pro-inflammmatory signals released from I/R myocardium largely mediates inflammatory response, including ROS generation, release of proteases, arachidonic metabolites, and chemokine [38]. The upregulation of cellular adhesion molecules promotes the recruitment and infiltration of neutrophils to injury sites [38]. Infiltration of neutrophil into ischemic cardiac tissue aggravates I/R injury and leads to infarct expansion, while removal of neurtrophil or drug inhibition of neutrophil activity has been shown to reduce infarct size in animal model [39, 40].

The activation of the HO-1 pathway plays a protective role in the myocardium during I/R injury. Transgenic mice that were heterozygous mutants for HO-1 gene disruption exhibited a deterioration of infarct size after myocardial I/R stress [41]. Conversely, cardiac specific overexpression of HO-1 has been shown to protect against I/R injury with improvement of contractile recovery and reduction of infarct size [42]. Myocardial HO-1 induction by acute treatment with the selective HO-1 inducer hemin has been shown to protect against myocardial I/R injury [43], while chronic HO-1 activation by prolonged administration of hemin also improved survival rate by exerting a potent antioxidant activity and disrupting multiple levels of the apoptotic and inflammatory cascade [44]. HO-1 activity is crucial for activation of the protective effect. Administration of bilirubin in cells has been shown to markedly reduce the cytotoxicity produced by oxidants [45]. Higher bilirubin levels are also related to decreases in lipid peroxidation and associated with alleviation from the risk of coronary artery disease in humans [7, 46]. Also important is that upregulation of HO-1 activity can resist diabetes-induced oxidative stress by increasing SOD and catalase activity [13]. Our study suggests that PLCA stimulation of HO-1 signaling resulted in the improvement of mnSOD expression and catalase activity, which may contribute to the attenuation of oxidative injury during I/R stress.

HO-1 expression is regulated by Nrf2 (NF-E2-related factor-2) transcription factor [17]. After stimulation, the free Nrf2 translocates to the nucleus, binds to response elements and activates HO-1 expression [47]. AKT, a potential target, is a key survival signaling pathway [48, 49]. AKT not only promotes cell survival, but also modulates Nrf2 as an upstream signaling molecule, and activates HO-1 to protect against oxidative stress [50, 51]. Inhibition of PI3K/AKT signaling by LY294002 reduced the elevation of Nrf2 levels and resulted in decreased binding to the promoter sequence of HO-1 [51], thus inhibiting activation of HO-1 expression [24, 52]. Therefore, the AKT pathway plays a vital role in HO-1 mediated antioxidant response. PLCA activated HO-1 through AKT signaling, where inhibition of AKT abrogated the protective effect of PLCA. The activations of AKT and HO-1 are correlated, since inhibiting one of the pathways affects the expression of the other. Indeed, HO-1 overexpression resulted in increased levels of AKT phosphorylation [50]. It is still unclear how HO-1 phosphorylates AKT and whether HO-1 inhibits AKT dephosphorylation, but our data favor the idea that AKT and HO-1 may operate in a positive feedback mechanism, whereby the level of HO-1 expression reciprocally augments AKT activation, which in turn increases HO-1 expression [50].

In conclusion, activating an endogenous antioxidant signaling pathway is more important due to the fact that the free radical scavenging behavior in vitro is not always correlated with a cytoprotection effect in vivo. PLCA exhibits considerable antioxidant properties that work by enhancing AKT/HO-1 signaling to increase mnSOD expression and catalase activity, which may combat I/R stress. These protective effects may represent the contributions of PLCA to cardiac function improvement. These findings provide new insights that may help improve therapeutic approaches using modified natural compounds to reduce oxidative stress from myocardial injury.
